# Visceral adiposity index and prognosis among patients with ischemic heart failure: Índice de adiposidade visceral e prognóstico em pacientes com insuficiência cardíaca isquêmica

**DOI:** 10.1590/1516-3180.2015.01452111

**Published:** 2016-05-13

**Authors:** Patrícia Vogel, Airton Stein, Aline Marcadenti

**Affiliations:** I BSc. Specialist in Clinical Nutrition, Institute of Education and Research, Hospital Moinhos de Vento (IEP/HMV), Porto Alegre, RS, Brazil.; II PhD. Professor, Department of Public Health, Universidade Federal de Ciências da Saúde de Porto Alegre (UFCSPA), Porto Alegre, RS, Brazil, Professor, Institute of Health Technology Assessment, Hospital Nossa Senhora da Conceição, Porto Alegre, RS, Brazil.; III PhD. Professor, Postgraduate Cardiology Program, Institute of Cardiology, Fundação Universitária de Cardiologia (IC/FUC), Porto Alegre, RS, Brazil, Professor, Department of Nutrition, Universidade Federal de Ciências da Saúde de Porto Alegre (UFCSPA), Porto Alegre, RS, Brazil.

**Keywords:** Anthropometry, Body mass index, Heart failure, Mortality, Obesity, abdominal

## Abstract

**CONTEXT AND OBJECTIVES:**

The obesity paradox has already been established in relation to heart failure, but it is not known which obesity indicator best reflects this phenomenon. The aim of this study was to evaluate the association between obesity indexes and mortality among patients with heart failure.

**DESIGN AND SETTING:**

Cohort study conducted in the Department of Cardiology of Hospital Nossa Senhora da Conceição (Brazil).

**METHODS:**

Clinical, demographic, socioeconomic, biochemical and anthropometric data on 116 patients aged 30 to 85 years with a diagnosis of heart failure were evaluated. Arm fat area, body mass index, body surface area, body adiposity index, lipid accumulation product (LAP) and visceral adiposity index (VAI) were calculated. Cox regression was used to perform survival analyses.

**RESULTS:**

At baseline, the individuals with ischemic heart failure who remained alive showed higher VAI (3.60 ± 3.71 versus 1.48 ± 1.58; P = 0.04) and a trend towards higher LAP, in comparison with the individuals who died. After an average follow-up of 14.3 months, ischemic heart failure patients who had VAI > 1.21 showed 78% lower risk of death (HR 0.12; 95% CI: 0.02-0.67; P = 0.02) and the Kaplan-Meier survival curves showed better prognosis for these individuals (P = 0.005; log-rank test).

**CONCLUSION:**

Our results suggest that VAI is a good predictor of better prognosis among ischemic heart failure patients.

## RESUMO

**CONTEXTO E OBJETIVOS:**

O paradoxo da obesidade já está consolidado na insuficiência cardíaca, mas não se sabe qual indicador de obesidade melhor reflete esse fenômeno. Este estudo teve como objetivo avaliar a associação entre índices de obesidade e a mortalidade entre pacientes com insuficiência cardíaca.

**TIPO DE ESTUDO E LOCAL:**

Estudo de coorte realizado no Departamento de Cardiologia do Hospital Nossa Senhora da Conceição (Brasil).

**MÉTODOS:**

Foram incluídos 116 pacientes com diagnóstico de insuficiência cardíaca de 30 a 85 anos. Dados clínicos, demográficos, socioeconômicos, bioquímicos e antropométricos foram avaliados. Área de gordura do braço, índice de massa corporal, área de superfície corporal, índice de adiposidade corporal, produto da acumulação lipídica e índice de adiposidade visceral foram calculados; regressão de Cox foi usada para realizar análises de sobrevida.

**RESULTADOS:**

No início do estudo, indivíduos com insuficiência cardíaca isquêmica que se mantiveram vivos mostraram valores mais elevados de índice de adiposidade visceral (3,60 ± 3,71 contra 1,48 ± 1,58, P = 0,04) e uma tendência para produto da acumulação lipídica maior quando comparados com os indivíduos que morreram. Após acompanhamento médio de 14,3 meses, os pacientes com insuficiência cardíaca isquêmica que apresentaram índice de adiposidade visceral > 1,21 tiveram 78% menor risco de morrer (HR 0,12; IC 95% 0,02-0,67, P = 0,02) e as curvas de Kaplan-Meier para sobrevida mostraram melhor prognóstico nesses indivíduos (P = 0,005, teste *log-rank*).

**CONCLUSÃO:**

Nossos resultados sugerem que o índice de adiposidade visceral é um bom preditor de melhor prognóstico em pacientes com insuficiência cardíaca isquêmica.

## INTRODUCTION

Heart failure is a complex clinical conditional and it is considered to be an important public health problem. Despite significant therapeutic advances, heart failure is highly associated with morbidity and mortality in both developed and developing countries.^1^

Obesity is a well-known risk factor for cardiovascular diseases, including heart failure.^2^^,^^3^^,^^4^ However, studies conducted among ischemic and non-ischemic heart failure patients have suggested that high adiposity indexes may be associated with better prognosis and longer survival than among patients with normal and lower weight.^5^^,^^6^ This association is known as the “obesity paradox” or “reverse epidemiology”.^7^^,^^8^

Most studies on the obesity paradox use body mass index to assess overweight and detect possible associations with the prognosis.^9^^,^^10^ Although body mass index is the most common and practical method for classifying obesity, there has been some discussion about whether it is indeed a good marker for patients with chronic diseases.^11^^,^^12^^,^^13^ Studies have been conducted with the aim of evaluating the prognostic value of other adiposity measurements such as waist circumference^14^ and body composition assessed through cutaneous skinfolds,^15^ dual-energy X-ray absorptiometry (DEXA) or bioelectrical impedance analysis^16^^,^^17^ among subjects with heart failure.

The lipid accumulation product index^18^ and the visceral adiposity index^11^ have been proposed as indirect measurements of visceral adipose tissue and have been correlated with cardiometabolic risk, cardiovascular disease and mortality in the general population.^19^^,^^20^ The body adiposity index is an alternative to the body mass index for determining the percentage of body fat,^21^ and neck circumference has been characterized as a pathogenic fat depot correlated with visceral adipose tissue and worse metabolic profile.^22^ However, none of these adiposity measurements has been tested among patients with ischemic or non-ischemic heart failure, within the context of the obesity paradox.

The purpose of this study was to evaluate associations shown by indexes of general and abdominal obesity in relation to mortality, among patients with ischemic and non-ischemic heart failure.

## OBJECTIVE

To evaluate associations shown by indexes of general and abdominal obesity in relation to mortality, among patients with ischemic and non-ischemic heart failure.

## METHODS

### Setting and study design

A cohort study was conducted between July 2011 and January 2013 in the Department of Cardiology, Hospital Nossa Senhora da Conceição (Porto Alegre, Rio Grande do Sul, Brazil), which provides healthcare within the National Health System that is used mostly by patients belonging to lower social classes. The research protocol was approved by the Research Ethics Committee of the Conceição Hospital Group (CEP-GHC no. 10-118) and all the patients signed a consent statement in order to participate.

A sample of 116 patients aged 30 to 85 years who were admitted to the Cardiology Unit of Hospital Nossa Senhora da Conceição due to complications and symptoms relating to decompensated heart failure (for instance: dyspnea, fatigue or edema), of New York Heart Association classes I-IV, was consecutively selected. Patients with significant complications relating to heart failure (coronary artery disease, cerebrovascular disease or severe renal impairment) within the last 6 months, individuals with acute coronary syndrome within the last 90 days, patients with valvular heart disease, candidates for myocardial revascularization surgery, patients with a history of cancer over the last two years and patients who were not on a condition in which anthropometric data could be verified (those with amputation of lower limbs or sequelae of stroke) were excluded.

At baseline, demographic data (age, sex and self-reported skin color) and information regarding education (years at school) and lifestyle characteristics (smoking, abusive alcohol consumption [≥ 30 g for men and ≥ 15 g for women] and physical activity) were collected by trained personnel (physicians, medical students and nutritionists) using a standardized questionnaire. Clinical data were obtained directly from the medical records or after medical evaluation: angina (stable or unstable) was diagnosed by a trained cardiologist in accordance with current guidelines through reviewing the patient’s electronic medical records;^23^ atrial fibrillation was also defined by a trained cardiologist in accordance with the guidelines and after cardiac auscultation;^24^ and blood pressure measurements were made using an aneroid sphygmomanometer with an adequately sized cuff around the arm circumference. Hypertension was defined as a diagnosis of systolic blood pressure ≥ 140 and/or diastolic blood pressure ≥ 90 mmHg, from a previous medical diagnosis or through use of antihypertensive pressure-lowering agents.^24^ Fasting blood glucose ≥ 126 mg/dl or glycated hemoglobin ≥ 6.5% or a previous medical diagnosis were used to detect patients with type 2 diabetes mellitus.^25^ Ejection fraction values, in percentages, were obtained from color Doppler-derived measurements on tissues, by means of transthoracic echocardiography, performed using the GE Vivid 3 system (General Electric, Norway).

Complete blood count, serum creatinine, HDL-cholesterol, serum triglycerides, glycated hemoglobin and fasting blood glucose were obtained from 10 ml venous blood samples from the patient, using standardized techniques at the hospital’s certified laboratory. Hematological parameters were detected using a Sysmex XE-5000 analyzer (Sysmex, Kobe, Japan); serum creatinine values were obtained through the kinetic colorimetric Jaffé method; HDL-cholesterol and triglyceride levels were detected by means of the colorimetric enzymatic method; glycated hemoglobin levels were examined through electrochemiluminescence; and fasting blood glucose values were obtained using the modified hexokinase enzymatic method.

After hospital discharge, the patients were contacted every six months by telephone to collect information about their vital status. All patients were contacted at least three times during the follow-up. In cases of death, this was confirmed from the medical records and/or the death certificate, which was brought to the researchers by a member of the family.

### Anthropometric parameters

Weight, height, arm circumference, neck circumference, waist circumference, hip circumference and triceps skinfold thickness measurements were obtained. Weight (kg) was measured with the patients wearing light clothes and barefoot, standing on weighing scales with scale divisions of 100 g (Filizola model 31, São Paulo, Brazil), and height was obtained using a stadiometer with scale divisions of 0.1 cm (Tonelli model E120 A; IN Tonelli SA, Santa Catarina, Brazil). Circumferences were measured with a non-elastic measuring tape. Arm circumference was obtained at the midpoint between the acromion and the olecranon, with the arm extended down the side of the body and the palm of the hand facing the thigh. Waist circumference was measured at the largest part between the waist and the thigh, while the subjects were wearing thin clothes. the narrowest part between the hips and the ribs. The triceps skinfold thickness was measured using a plicometer at the midpoint between the acromion and the olecranon with the arm extended down the side of the body and the palm of the hand facing the thigh.^26^ Neck circumference was obtained from the midpoint of the neck.^27^

Adiposity measurements were calculated as follows:

- Arm fat area (cm^2^) = (arm circumference (cm)*[triceps skinfold thickness (mm)/10]/2) - (3.14*[triceps skinfold thickness (mm)/10]^2^/4). The 90^th^ percentile was taken to be the cutoff point for obesity.^28^
- Body mass index (kg/m^2^) = weight (kg)/height^2^ (m). The cutoff point for obesity was taken to be ≥ 30 kg/m^2^.^29^
- Body surface area (m^2^) = (0.007184*(height (cm))^0.725)^*(weight (kg)^0.425)^.^30^
- Body adiposity index (%) = (hip circumference (cm)/(height)^1.5^) - 18.^21^
- Lipid accumulation product index (cm.mmol.l) = (waist circumference (cm) - 65)* triglycerides (mmol/l) for males; and lipid accumulation product index = (waist circumference (cm) - 58)* triglycerides (mmol/l) for females.^18^
- Visceral adiposity index = (waist circumference (cm)/(39.68 + (1.88*body mass index))*(triglycerides/1.03)*(1.31/HDL) for males; and visceral adiposity index = (waist circumference (cm)/(36.58 + (1.89*body mass index))* (triglycerides/0.81)*(1.52/HDL) for females.^11^

### Statistical analysis

Analyses were performed in accordance with the etiology of heart failure (ischemic or non-ischemic), in order to explore the possible role of the etiology in relation to anthropometric indexes and mortality. Data were expressed as mean ± standard deviation or as frequencies (%). Student’s t test (parametric variables), Wilcoxon and Mann-Whitney tests (nonparametric variables) and Fisher’s exact test (categorical variables) were used for comparisons. We used the log-rank test, Kaplan-Meier curves and Cox regression model for survival analyses. All anthropometric data were categorized as percentiles, and the reference category was defined as ≥ 25^th^ percentile. The lipid accumulation product index, visceral adiposity index and body adiposity index do not have specific cut-off points, but all the adiposity indexes were standardized at the 25^th^ percentile in order to maintain comparability.

Analyses were performed using the Statistical Package for the Social Sciences (SPSS) software, version 17.0 (SPSS, IL, USA). For each analysis, α-level = 0.05 was considered significant, and 95% confidence intervals were shown.

## RESULTS

During the follow-up period (mean duration of 14.3 ± 10.2 months), the general mortality rate was 20.6%. It was 30% (six deaths) among the patients with ischemic heart failure and 18.8% (18 deaths) among those with non-ischemic heart failure. No statistically significant correlation was identified between the heart failure etiology and mortality rate (P = 0.3).

The patients’ mean age was 61.8 ± 12.3 years; 62.1% were males; 71.6% were whites; the mean number of years of school attendance was 5.0 ± 3.4; 12.1% were current smokers; and 8.6% presented abusive consumption of alcohol. According to the body mass index, 41 (35.4%), 36 (31%) and 39 (33.6%) presented, respectively, normal weight, overweight and obesity. Regarding the New York Heart Association functional class, 88 (75.9%) were in classes III-IV. The mean ejection fraction was 40.6 ± 14.8%. The prevalences of hypertension and type 2 diabetes mellitus were 77.6% and 32.8% respectively.

Table 1 shows the patients’ demographic and clinical characteristics, stratified according to heart failure etiology and survival. Among the patients with ischemic heart failure, there were no differences regarding age, ethnicity, New York Heart Association classification, ejection fraction, systolic and diastolic blood pressure, hemoglobin levels, diagnoses of hypertension, angina, atrial fibrillation or type 2 diabetes mellitus, or the number of drugs in use according to survival status (dead or alive). However, there was a trend for serum creatinine levels (P = 0.07). Among the individuals with non-ischemic heart failure, only hemoglobin levels were significantly lower in the group of patients who died (P = 0.01).

**Table 1.**
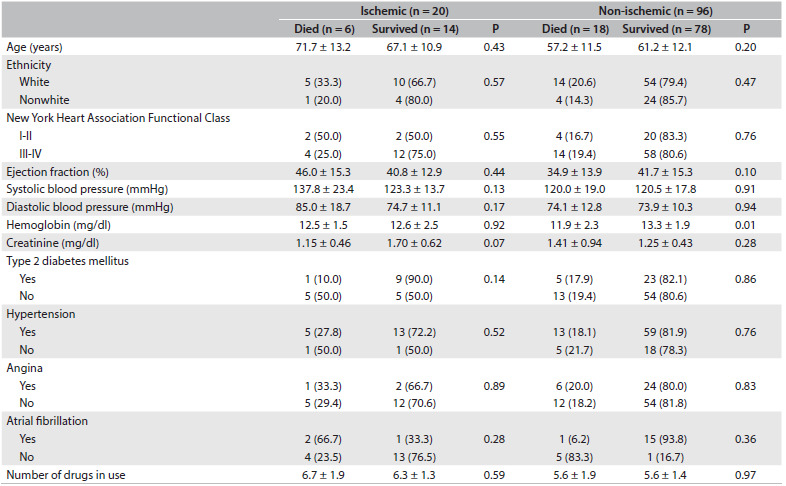
Demographic and clinical characteristics of the sample [mean ± SD or n (%)]

SD = standard deviation.

Regarding the main drugs used within the hospital setting, 101 patients (87.1%) were being treated with loop diuretics, 93 (80.2%) with beta blockers, 71 (61.2%) with platelet anticoagulant, 68 (58.6%) with angiotensin-converting-enzyme inhibitors, 55 (47.4%) with statins, 49 (42.2%) with potassium-sparing diuretics, 46 (39.7%) with digitalis, 39 (33.6%) with oral anticoagulants, 18 (15.5%) with angiotensin receptor blockers and 13 (11.2%) with calcium channel blockers.

The adiposity indexes according to heart failure etiology and survival status are described in Table 2. There were no significant differences in most of the anthropometric parameters, except for the visceral adiposity index and a trend for lipid accumulation product index values among individuals with ischemic heart failure, which were both higher among survivors than among patients who died (3.60 ± 3.71 versus 1.48 ± 1.58; P = 0.04 for visceral adiposity index; and 70.49 ± 50.73 versus 35.49 ± 32.62; P = 0.08 for lipid accumulation product index).

**Table 2.**
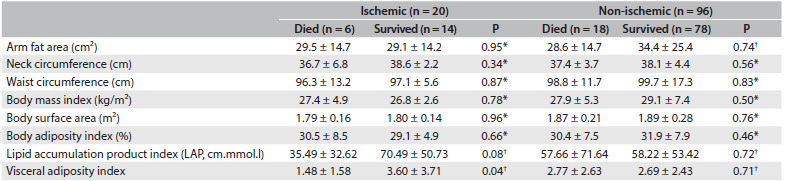
Anthropometric characteristics of the sample (mean ± SD)

SD = standard deviation. ^*^Student’s t test (parametric variables), taking P < 0.05 (5%) to be significant; ^†^Wilcoxon and Mann-Whitney tests (nonparametric variables) for comparison, taking P < 0.05 (5%) to be significant.

**Figure 1.**
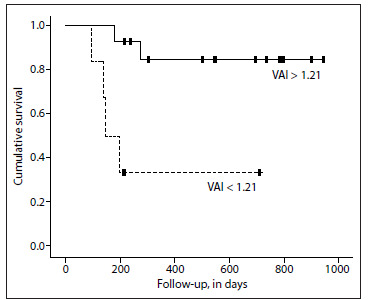
Kaplan-Meier survival curves plotted for patients with ischemic heart failure etiology (VAI: visceral adiposity index).

Survival analyses performed using the Cox regression model showed that higher values for the visceral adiposity index (> 1.21) were related to better prognosis (HR 0.12; 95% CI: 0.02-0.67; P = 0.02) among patients with ischemic heart failure etiology. Kaplan-Meier survival curves plotted for patients with ischemic heart failure etiology showed that survival prognosis improved with visceral adiposity index values > 1.21 (P = 0.005 detected by means of the log-rank test) (Figure 1). Other anthropometric data did not show any relationship with prognosis in survival analyses.

## DISCUSSION

To our knowledge, this is the first study on patients with heart failure to have evaluated non-traditional adiposity indexes as prognostic markers. Moreover, our study showed an inverse relationship between the visceral adiposity index as an abdominal obesity index and mortality, among patients with ischemic heart failure, and we were unable to find associations between general adiposity indexes (body adiposity index and body mass index) and survival in our population.

The obesity paradox has been widely studied, but controversy remains with regard to the best adiposity index for making the prognosis among individuals with ischemic and non-ischemic heart failure. A meta-analysis that evaluated nine studies and included 28,209 individuals with heart failure showed that overweight (body mass index 25-29.9 kg/m^2^) and obesity (body mass index ≥ 30 kg/m^2^) detected by means of body mass index cutoff points were associated with lower cardiovascular and all-causes mortality.^8^ Among individuals with heart failure and type 2 diabetes mellitus, body mass index 25-30 kg/m^2^ was also correlated with lower mortality.^31^ On the other hand, obesity class III (body mass index ≥ 40 kg/m²) was correlated with higher mortality rates.^32^^,^^33^

Although higher body mass index (≥ 25 kg/m^2^) has been commonly correlated with a favorable prognosis, a meta-analysis including 12 studies and 6,142 individuals with acute decompensated heart failure showed that higher body mass index was associated with lower mortality, particularly among very elderly people (age > 75 years), individuals with reduced ejection fraction (< 50%), people without type 2 diabetes mellitus and cases of newly diagnosed heart failure. This suggested that aging, heart failure severity/chronicity and metabolism might explain the obesity paradox regarding use of the body mass index as an adiposity measure.^34^ Moreover, among obese individuals with heart failure, symptoms may appear earlier, and therefore the diagnosis and treatment can take place at earlier stages of heart failure.

We were unable to find any association with body mass index and mortality according to heart failure etiology, and other authors have shown conflicting results. Among chronic heart failure patients, after a mean follow up of 30.7 months (annual event rate 6.0%), the ischemic heart failure overweight subgroup had a lower survival rate than the non-ischemic heart failure overweight subgroup; however, normal weight and obese individuals showed no difference regarding mortality and heart failure etiology.^5^ Among individuals with chronic heart failure classes II and III, the obesity paradox was only observed in patients with non-ischemic heart failure after adjustment for age, sex, New York Heart Association functional class, ejection fraction, comorbidities and treatment.^6^ These studies were conducted among individuals who were in a stable condition and receiving outpatient treatment, whereas our patients were evaluated in a hospital setting and had higher prevalence of New York Heart Association functional classes III and IV. Thus, it can be suggested that heart failure severity strongly contributes towards mortality independently of heart failure etiology and body mass index values.

Using body mass index as a body fat measurement may lead to misclassification of around 40% of patients with heart failure^35^ and the usefulness of body mass index for evaluating body composition in these subjects has been questioned.^10^^,^^12^ Body mass index does not detect the total amount of body fat^12^^,^^35^^,^^36^ and its classification does not vary according to sex, age or ethnicity.^36^ As well as in cases of heart failure, the obesity paradox has been shown in other cardiovascular disorders such as coronary artery disease.^37^ Grade I obesity has not been associated with mortality among the general population.^38^ However, body mass index has a good correlation with lean mass among individuals with coronary artery disease^13^ and also in the general population.^12^ Thus, the poor diagnostic performance of body mass index in discriminating fat and lean mass and also in assessing body fat content could explain the obesity paradox. Furthermore, the prognostic value of the body mass index in specific situations, such as athletes and patients with fluid accumulation, is also limited.^12^

The effects of body fat detected through other assessment measurements such as cutaneous skinfolds, bioelectrical impedance analysis and DEXA on the obesity paradox have also being studied, given the limitations of the body mass index. Increased body fat detected through DEXA has been correlated with lower mortality among cases of heart failure.^35^ However, these techniques are not always available and may be expensive, thus restricting their use in clinical practice. The body adiposity index is a simple alternative to the body mass index for detecting percentage body fat; however, we were unable to detect any association between higher body adiposity index and survival among cases of heart failure. It has been suggested that using the body adiposity index as an indicator of overall adiposity is likely to produce biased estimates of percentage body fat, in which the errors may vary according to sex and level of body fatness. Thus, estimates based on the body adiposity index may not be more accurate than those based on the body mass index.^39^

The prognostic value of waist circumference as an abdominal obesity index has also been studied among patients with heart failure. Interestingly, higher waist circumference values have been shown to have a protective role regarding mortality rates among patients with heart failure.^7^^,^^14^ Increased waist circumference and normal body mass index may reflect lower levels of physical activity among healthy individuals, with consequently higher levels of fat mass and lower lean mass.^40^ However, waist circumference has been correlated with both lean and fat mass detected by means of bioelectrical impedance analysis among individuals with heart failure, thus suggesting that both compartments are associated with a better prognosis among these patients.^17^

Waist circumference cannot distinguish subcutaneous adipose tissue from visceral adipose tissue, but it seems to be more strongly associated with subcutaneous fat, especially among overweight individuals.^41^ Higher levels of visceral fat have been correlated with higher levels of inflammatory cytokines such as tumor necrosis factor-α, which have catabolic effects on lean mass and may contribute towards cardiac cachexia.^42^^,^^43^ However, tumor necrosis factor-α receptors are highly expressed in subcutaneous adipose tissue,^44^ and heart failure patients with an enlarged waist may be protected from the negative impact of increased levels of tumor necrosis factor-α through production of higher levels of these receptors, compared with patients with normal weight or who are underweight.^7^ Since weight lost is associated with lower survival in situations of heart failure,^45^ patients with greater severity of heart failure who also have excess body fat (including both the subcutaneous and the visceral compartment) may have greater metabolic reserves and be more resistant to the increased catabolic burden.^46^

Obese individuals and patients with ischemic heart disease have higher levels of lipoproteins such as cholesterol and chylomicron, and this contributes towards higher bacterial lipopolysaccharide levels, thereby stimulating release of pro-inflammatory cytokines.^47^^,^^48^ Anthropometric and serum lipid values are needed in order to calculate both the lipid accumulation product index and visceral adiposity index. However, the formula for the visceral adiposity index includes two biomarkers that relate to lipoproteins (HDL-cholesterol and triglycerides) and two obesity indexes that are associated with lean mass and subcutaneous fat (waist circumference and body mass index), thus suggesting that instead of being a “visceral adipose function” as proposed originally,^11^ the visceral adiposity index might reflect an “excess of weight function” among patients with heart failure.

This exploratory study may have been influenced by the small sample size, which will have conferred higher variability, and it may have lacked power to detect some associations, such as the lipid accumulation product index and death. Thus, some of our results may have been due to chance. Furthermore, we did not construct an adjusted multivariable model to detect independent associations between anthropometric indexes (especially the visceral adiposity index) and mortality. Our patients were relatively young and were predominantly in New York Heart Association classes III-IV, without a preserved ejection fraction, which may have limited the extrapolation of our results. On the other hand, the data collection was prospective, which considerably improves data quality. In addition, important prognostic factors were considered in our analysis.

## CONCLUSION

We suggest that the visceral adiposity index may be a good predictor of mortality among patients with ischemic heart failure. However, further studies are needed in order to confirm these results.
